# Modularising ontology and designing inference patterns to personalise health condition assessment: the case of obesity

**DOI:** 10.1186/s13326-016-0049-1

**Published:** 2016-05-04

**Authors:** Aleksandra Sojic, Walter Terkaj, Giorgia Contini, Marco Sacco

**Affiliations:** Institute of Industrial Technologies and Automation (ITIA), National Research Council (CNR), Via Bassini 15, Milan, Italy

**Keywords:** Obesity, Ontology modularisation, Personalised inference, Physical constitution, Physical activity, Nutritional habits, Healthy lifestyle, Person, Teenager

## Abstract

**Background:**

The public health initiatives for obesity prevention are increasingly exploiting the advantages of smart technologies that can register various kinds of data related to physical, physiological, and behavioural conditions. Since individual features and habits vary among people, the design of appropriate intervention strategies for motivating changes in behavioural patterns towards a healthy lifestyle requires the interpretation and integration of collected information, while considering individual profiles in a personalised manner. The ontology-based modelling is recognised as a promising approach in facing the interoperability and integration of heterogeneous information related to characterisation of personal profiles.

**Results:**

The presented ontology captures individual profiles across several obesity-related knowledge-domains structured into dedicated modules in order to support inference about health condition, physical features, behavioural habits associated with a person, and relevant changes over time. The modularisation strategy is designed to facilitate ontology development, maintenance, and reuse. The domain-specific modules formalised in the Web Ontology Language (OWL) integrate the domain-specific sets of rules formalised in the Semantic Web Rule Language (SWRL). The inference rules follow a modelling pattern designed to support personalised assessment of health condition as age- and gender-specific. The test cases exemplify a personalised assessment of the obesity-related health conditions for the population of teenagers.

**Conclusion:**

The paper addresses several issues concerning the modelling of normative concepts related to obesity and depicts how the public health concern impacts classification of teenagers according to their phenotypes. The modelling choices regarding the ontology-structure are explained in the context of the modelling goal to integrate multiple knowledge-domains and support reasoning about the individual changes over time. The presented modularisation pattern enhances reusability of the domain-specific modules across various health care domains.

## Background

Overweight and obesity are estimated to result in the deaths of about 320 000 people in western Europe every year [1]. The prevalence of obesity among children and adolescents motivated public health organisations to promote a healthy lifestyle by specifically engaging children [1, 2] and adolescents [1, 3, 4]. The initial activity towards the engagement of individuals consists in the design of a scientifically informed strategy, i.e. the development of a model that defines the key features associated with obesity. Capturing this knowledge is a complex task since it often requires understanding intertwined relations between various phenotypic parameters and socio-behavioural aspects of lifestyle [5]. Availability of technological devices that can register data associated with physical constitution, physiology, behavioural habits related to physical activity, and nutrition enables the acquisition of more specific insight into physical characteristics of individual people and their behavioural patterns [6]. The interpretation and understanding of the acquired data involves multiple domains of knowledge and the analysis of heterogeneous information. The relevant data need to be collected, organised, and integrated in order to provide a feedback that is appropriate to a specific personal profile.

The task of representing personal profiles in a model that integrates diverse kinds of data provided by various sources motivates the employment of Semantic Web technologies [7]. In particular, ontologies are recognised as a convenient approach to deal with complex and heterogeneous information across various domains [8–10], enabling data interoperability [11, 12] and also knowledge generation via reasoning [8, 9]. Unlike some alternative modelling approaches (i.e. relational databases), ontology models incorporate semantics, formalising and explicating shared understanding of a domain that can be easier to reuse across various applications (for the comparison of ontologies and relational databases see [13–16]).

Several studies report the use of ontology and semantic technologies to target obesity (e.g. [2, 17, 18]). Scala et al. [18] present an e-Knowledge platform, based on a Web Ontology Language (OWL) [19] ontology and Semantic Web Rule Language (SWRL) [20] rules, classifying individuals according to the obesity level and certain medical conditions (Sarcopenia, Hypertension, Dyslipidemia, Diabetes, Insulin resistance, Metabolic syndrome). Arash et al. [17] and Addy et al. [2] present the preliminary stage of an ontology designed to support a knowledge-based infrastructure, promoting healthy eating habits and lifestyles. In particular, Addy et al. [2] aim to support the ontology-based decision making across multi-stakeholder partnerships (MSPs) of the Quebec community involved in the management of childhood obesity.

The relevant literature addressing the issues related to obesity mostly refers to specific scenarios, focusing either on adults with certain diseases [18] or on children within a local community context [2, 17].

Since the public health concerns related to obesity [1] include various scenarios (e.g. diverse social, geopolitical and age groups, etc.), a generic model that would cover diverse knowledge-domains and application-contexts related to obesity would be beneficial as it could exploit the full potential of ontology-based modelling that goes beyond single application (see e.g. [11, 21]).

In order to face the need to model complex and diverse aspects of obesity-related knowledge, this paper in particular deals with:

- *Formalisation of generic knowledge related to obesity*.
- *Specialisation of the generic model into a teenager tailored model*.
- *Modularisation to support integration of generic and specific knowledge.*
- *Changes of personal features over time*.
- *Automatic inference of personal health status that is relevant for obesity assessment and prevention.*

The possibility of having both a general and a specific model is achieved by adopting a modular design strategy. The core ontology module specifies certain generic classes that are applicable to any human being and a generic characterisation of individual health conditions. The domain-specific ontology modules (applicable to any person) provide obesity-related classifications. The core module as well as the domain-specific ontology modules are formalised in OWL (see the following sections). The modules specifying sets of rules are modelled in SWRL and explicitly provide reference values that support inference and classification of personal profiles for the population of teenagers. In particular, the ontology design is driven by the need to track changes in health condition over time (i.e. the issue that was not addressed by [17, 18]).

Thus, the ontology model presented in this paper addresses the problem that was only partially addressed in the models previously described in the literature (i.e. [2, 17, 18]) as it faces modelling of *personal profiles* on a *generic* level to support *specific inference* within a *comprehensive* ontology model of *the obesity-related knowledge*. The developed ontology formalises information about obesity-related human features, enables reasoning, and enables information flow and interoperability between the technological tools and platforms employed to monitor the changes of health status, behaviour, and nutritional habits of humans in general, and adolescents in particular.

The development of this ontology was initiated within the European research project named PEGASO [22] whose main goal is the enhancement of self-awareness and motivation of adolescents towards a healthy lifestyle [3, 23, 24]. Like some other initiatives (e.g. [2, 17]), the project is driven by the public health concerns aiming at the decrease of obesity-related risks to health [3, 25]. The target population is represented by the future adults whose behavioural habits at an early age can significantly impact their health status on a life-long horizon [23]. The project includes several research initiatives and interventional strategies such as the development of serious games [3, 26] that should promote a healthy lifestyle, the design of a life companion [24], the use of wearable gadgets equipped with sensors to monitor health status [3, 24], the design of mobile applications such as an e-diary used to record dietary habits, etc. [3, 24].

In the following sections we first outline the theoretical and practical context that is relevant in explaining and justifying the decisions taken during the ontology design phase. We discuss the modular structure of the ontology as related to the methodological approach that considers ontology-design from two perspectives:

(1) task dependent modelling that faces a particular application scenario and (2) extrapolation of general modelling patterns that can be used in a broad context that goes beyond a single application task. We specifically describe an ontology module that captures the physical domain and classifies health conditions based on the assessment of body constitution. In the second part of the paper we present the inference patterns that are used in the current version of the ontology. In order to exemplify the employment of reasoning patterns, we provide the case of reasoning over a personal assessment of health condition by combining OWL and SWRL rules. The concluding remarks outline some advantages of the modular structure and inference patterns, discussing the potential of their reuse in other application scenarios.

## Methods

The aim of this section is to explain and justify the representational choices employed in the ontology design. The initial step in the ontology development includes a multi-disciplinary analysis that considers a person as a dynamic agent who is constantly changing in their interaction with their environment. Thus, the methodology for the ontology design includes a detailed specification of the modelling domain(s), goal(s), and context of current scientific knowledge, i.e. theories used to define the key concepts relevant for capturing obesity-related knowledge in a comprehensive manner. Since the domain problem covers several fields of knowledge that are related to the problem of obesity, the specific fields are identified as distinct sub-domains of knowledge. The identified fields are later used to structure knowledge into the dedicated ontology modules, each of which can exist independently as the modules capture field-specific aspects of human features that are relevant for the modelling task and are also applicable to a wide scope of related scenarios. The methodology is in line with the tradition that considers *ontology* as an engineering artifact that is useful to model some aspects of the world. In other words, we accept the position that in Artificial Intelligence Systems, “what exists” is what can be represented ([27], p. 908–909).

### A preliminary study of a cross-disciplinary approach

The preliminary analysis of domain-knowledge, as presented below, is relevant for (1) the specification of the ontology goal and scope, (2) the methodology for the ontology design, and (3) the justification of the representational choices regarding the study of obesity and its prevention. The impact of cross-disciplinary studies on the ontology design are considered in the context of background knowledge and theories that the ontology needs to capture formally. The explication of the design-rationales aims at reducing opacity of the developed ontology and increasing its re-usability (c.f. [28], p. 222).

While dealing with the problem of obesity, its characterisation and prevention, it is important to consider several factors such as physical (in)activity, physiological (dys)function, (un)healthy eating habits, social and psychological problems [3]. In some cases, one of these aspects can be more decisive than the others causing overweight or obesity, whereas in other cases the overweight-condition (or a related disease) is the result of a combination of several factors. In order to identify and, potentially, modify the most relevant factor(s) or a specific habit of an individual that increases the likelihood of developing an overweight-condition, it would be optimal to consider one’s current state from the perspective of a comprehensive model that captures the features of a human being as a whole [3]. Such a model can be understood as an abstract representation that aims at integrating the cross- disciplinary knowledge of humans in a broad context.

Lafortuna et al. [5], Guarneri et al. [3], Caon et al. [23] and Carrino et al. [24] carried out multidisciplinary studies to address the issue of obesity and its prevention via employment of smart devices and persuasive technologies [23]. The studies considered intertwined relationships between human individuals and their environment. In particular, the results of the studies on physical, physiological, and behavioural aspects of human phenotypes provided a comprehensive model, i.e. the so-called *Virtual Individual Model (VIM)* [5, 23] that is meant to be a theoretical framework to deal with obesity prevention. The VIM identifies the key components that influence the *health status* of a person with reference to overweight and obesity, focusing on adolescents in particular. Since the VIM captures obesity-related knowledge by common representational means, e.g. natural language definitions, tables and graphs (readable to competent human experts), the presented information was not specified in a formal language. In other words, the VIM lacks a formal semantics and explicitness that would disambiguate its terminological and ontological assumptions in order to structure the concepts and relations in a comprehensive and machine-readable form.

An elaboration of the contents of the VIM led to the identifications of the key targets of the ontology-model: (1) capture health conditions of individual teenagers; (2) detect personal obesity-related risk factors; and (3) optimise the information structure in order to provide a personalised feedback that can motivate behavioural changes towards a healthy lifestyle.

After the identification of the *ontology goal*, the next step in the ontology design was to partition the modelling domain by specifying the most relevant fields of knowledge that can be formalised as *independent sub-domain ontologies*, i.e. the modules that can be later *integrated* into the final ontology model (see the following section).

The identification of knowledge sub-domains started with the analysis of the VIM that characterises human individual on three levels:

(i) *the physical-physiological level* (see ‘Physical Status’ in [23], p.1812),
(ii) *the nutritional level* (see ‘Dietary Habits’ in [23], p.1813), and
(iii) *the psycho-social level* (see ‘Psychological Status’ in [23], p.1812).

Each of these three levels of the VIM addressed the problem of obesity and its prevention from diverse disciplinary perspectives, thus allowing the domain specialists to contribute with their expertise to a comprehensive view on the problem. However, since we aim at characterising the knowledge sub-domains as *distinct, coherent and complementary segments of knowledge*, the characterisation of the levels (i-iii) is insufficient as it lacks a clear demarcation criterion necessary for the development of independent ontology modules. For instance, level (ii) includes the characterisation of dietary habits and as such it partly intersects with level (iii), which also (from another perspective, i.e. psychological) aims at targeting behavioural habits, e.g. fruit and vegetable intake. On the other hand, level (i) deals with both physical and physiological aspects that are closely related, but nonetheless (ontologically) distinct. In addition, the physical description in (i), besides body constitution, also includes the characterisation of habits such as *physical activity* that actually represents a *behaviour*. As such, the characterisation of physical activity captures features that are distinct from those used in the description of some physical parameters related to body constitution. For the requirements of an ontologically clean and coherent model that follows proper classification criteria [11, 29, 30], a more specific distinction of the modelling-domain and its sub-domains is required.

The task at hand is to specify sub-domains of knowledge in a way that can support a sustainable ontology development, thus following a coherent modularisation approach. Accordingly, we define and combine the topic-centred and discipline-oriented demarcation criteria, where by *discipline* we consider any field of study that is covered by the current educational system (see e.g. ISCED: International Standard Classification of Education [31]). On the other hand, the topic-centred cri- terion considers not merely a topic of study addressed by some discipline, but the features of the objects targeted by some study are also taken into account according to the OntoClean methodological perspective [29]. Thus, the criterion distinguishes on a meta-level kinds of objects that are targeted by the study (i.e. *meta-topic*). The identification of a meta-topic can be illustrated by the previous example of *physical activity* and *physical features* of humans that can be the topics of study addressed by psychologists, nutritionists, general practitioner, and so on. As a selected topic of study might target ontologically distinct objects of interest, we used a meta-topic characterisation to discriminate between static and dynamic parameters, features changeable over time vs. rigid features, etc. For instance, while a living being will necessarily have weight and height, their values will change over time. Likewise, the date of birth can be considered as a rigid parameter bound for a person, while the age of an individual changes over time. Also the characterisation of *physical activity* might be considered as a topic that includes the description of physical features, but from the meta-topic point of view the description of the activity captures *behaviour* and not some static *physical features*. The description of *physical features* might complement the description of *physical activity*, but the two concepts have different meanings as they capture diverse aspects of the physical reality. Thus, the demarcation of the topic of interest was performed according to an onto-sensitive approach that was used jointly with the disciplinary criterion to define the ontology modules, as described in the following section.

### Ontology modularisation

Ontology modularisation is recognised as an important topic especially regarding the implementation, maintenance, and reuse of ontology [32–34]. Despite the fact that modularisation plays a significant role in ontology engineering, there is no universally accepted methodological approach to modularisation [35, 36]. Some approaches focus on logical criteria (see e.g. [37]), whereas others address the issue of modularisation from a broader perspective (see e.g. [35, 36]) arguing that the choice of a modularisation technique and methodological approach actually depends on the particular requirements defined by the modelling goal and the application scenario.

Even so, the opportunity to modularise an ontology already in its early developmental stages provides numerous advantages related to its evolution, maintenance and reuse.

**Fig. 1:**
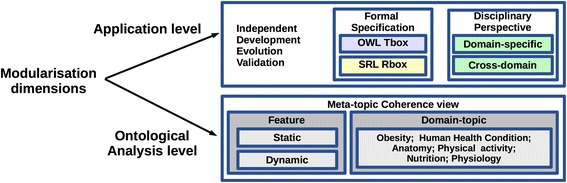
The Multidimensional Modularisation Methodology (MMM): the modularisation dimensions identified to support the ontological coherence on the theoretical level and sustainability on the application level (i.e. independent development, evolution, and validation)

Concerning the most appropriate modularisation strategy for the task at hand as well as for potential future applications [28, 32, 38, 39], several criteria were combined to define the modules, as outlined in Fig. 1.

First, the linearisation methodology for robust modular implementation of ontologies [32] is taken into account by adopting the following design criteria:

- the ontology modules are identified and separated from the whole;
- ontology maintenance is enhanced by enabling independent work on single modules;
- modules can evolve independently and new modules can be added with minimal side effects;
- the differences between different domain-specific categories are represented explicitly, thus enabling both human understanding and formal machine inference.

Furthermore, the *validation criterion* ([35], p.69) and the *domain coverage criterion* ([35], p.74) are taken into account in order to enable the independent validation of defined modules by different experts (See *Disciplinary Perspective* criterion in Fig. 1). Besides the fact that multiple fields of knowledge must be captured and validated independently, the intended formalisation in two modelling languages (i.e. OWL and SWRL) requires the language-specific validation [20] that motivates the separation of the segments formalised in OWL and the segments formalised in SWRL (see *Formal Specification* criterion in Fig. 1).

Finally, the main ontology structure is designed according to the *Multidimensional Modularisation Methodology (MMM)* (Fig. 1) that identifies the following criteria:

(a) the criterion of *disciplinary perspectives*; (b) the *meta-topic* coherence view, i.e. the criterion used to define (b1) the specific *Topic* that should be captured within an ontology module in an onto-sensitive and coherent manner, thus narrowing down the scope of a disciplinary perspective and domain coverage; and (b2) *features* that can be either dynamic or static (see the previous section). The *meta-topic* view is also used to specify the scope of (c) the *integrative-view* criterion that is used to identify common concepts shared across-domains, thus supporting the inter-module integration (e.g. intersecting the *Cross-domain* criterion and *Human Health Condition* topic – the example that will be discussed in the Results section).

**Fig. 2:**
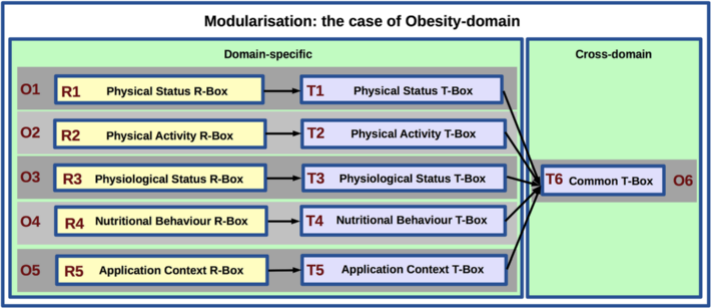
Ontology modularisation in the case of the Obesity domain: Applying The Multidimensional Modularisation Methodology (MMM) to identify specific ontology modules

Figure 2 illustrates the application of MMM to the Obesity domain, where criterion (a) was crucial in defining domain-specific modules (see O1-O5 with the segments T1-T5 and R1-R5), criterion (c) was particularly relevant in defining cross-domain modules (see T6 in Fig. 2), whereas criterion (b) had an important role in the characterisation of all the modules, providing an ontologically coherent structure. In addition, the *validation* criterion focused on *Formal Specification*, jointly with (b), is used to distinguish the ontology segments modelled in OWL (T1-T6) from the rule segments modelled in SWRL (R1-R5) (see Figs. 1 and 2). The resulting ontology consists of the following modules:

- (*O*_1_) *PhysicalStatus* that captures physical features of the human body.
- (*O*_2_) *PhysicalActivity* that captures the physical behaviour and habits.
- (*O*_3_) *PhysiologicalStatus* that captures certain physiological parameters.
- (*O*_4_) *NutritionalBehaviour*, capturing nutritional habits and behaviour.
- (*O*_5_) *ApplicationContext* that specifies the contextual information relevant for potential application scenarios, e.g. geographical location.
- (*O*_6_) *Common* module captures cross-domain information to support the interoperability across the modules (O1-O5).

**Fig. 3:**
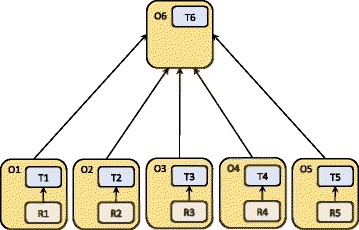
The modular architecture supporting independent ontology development, validation, use, and evolution. The sub-domain ontologies (O1-O5) are composed of domain-specific TBox and RBox modules. Module O6 consists of a TBox that is common to all other modules

Figure 3 presents the modular *architecture* designed to support independent development, validation, use, and evolution of the domain-specific ontology modules. The *Common* module (O6), formalised in OWL, consists of a TBox that provides terminological contents and the most generic specification of classes and relations that are common for all other modules (O1-O5). On the other hand, the domain-specific ontology modules (O1-O5) are composed of a TBox component formalised in OWL (see T1-T6) and an RBox component containing only domain-specific SWRL rules (see R1-R5) specified as extension of the corresponding TBox modules. In particular, the following RBox modules are used for personalised inference and classification of individuals within the target population of teenagers:

- (*R*_1_) *PhysicalStatus* Rules - used for personalised assessment of health conditions as based on the obesity classification.
- (*R*_2_) *PhysicalActivity* Behaviour Rules - used for the assessment related to behavioural habits, e.g. sedentariness.
- (*R*_3_) *PhysiologicalStatus* Rules - related to the assessment of health conditions based on physiological parameters, e.g. metabolism rate.
- (*R*_4_) *NutritionalBehaviour* Rules - used for the assessment of nutritional characterisation of individuals as based on the food and drink intake, e.g. breakfast skipper.
- (*R*_5_) *ApplicationContext* Rules - used for the context-dependent assessment to characterise conditions that vary across socio-cultural contexts, e.g. modifying the assessment based on the information about geographical location.

These RBoxes define the rules based on current knowledge of the relationships between the captured parameters and the reference values acquired by the team of experts, the World Health Organisation (WHO) reference tables for the population of adolescents [40], and the most recent literature in the domain of interest that is provided in the ontology annotation. On the other hand, the TBoxes are modelled to be more stable and population independent. By keeping TBox and RBox separate, the ontology validation, maintenance, reusability, and evolution are enhanced. For example, the rules in the RBox are defined according to the current state of knowledge that defines cut-off values used in classifying a teenager as obese, over-weight etc. In case the state of knowledge changes (or a target population changes), any change in cut-off values specified in the rules will not impact the ontology as a whole and modifications can be made only within the rules that contain the up-dated values. In addition, separating RBoxes specified in SWRL from the TBoxes specified in OWL enables independent and the language-specific validation [20] of the OWL and SWRL ontology segments.

## Results and discussion

This section focuses on the ontology content. While presenting the modelling patterns, we describe the *Common* and *PhysicalStatus* ontology modules, specifying the most relevant body features as related to measurements and to several other classes of health conditions that are used to define the obesity-related status and potential risk factors.

### Capturing normative concepts: assessment of obesity as a health condition

In general terms, a description of a person (e.g. a teenager) via some structural, functional, and behavioural characteristics is actually capturing aspects considered to be relevant to describe his/her phenotype (that might be a teenager-specific phenotype). The main focus is on the phenotypic features describing the class to which a person belongs as determined by the characterisation of his/her physical and behavioural features [41]. Thus, we consider that a person’s phenotype belongs to the class *obese* based on his/her characteristics, description of which (despite of individual variations) fits to the description of an obese phenotype that is typical for every person of a certain *gender* and *age* range. We define *typical* features of an *obese* phenotype in terms of a conventional agreement at the current stage of knowledge. The reference system that we use to characterise the physical features of an obese phenotype is provided by the World Health Organisation [25] and it includes the age- and gender-specific ranges of values, e.g. body mass index of teenager (see [40]). Moreover, we treat the description of body constitution as a specific characterisation of phenotype that is associated with *health condition*.

**Fig. 4:**
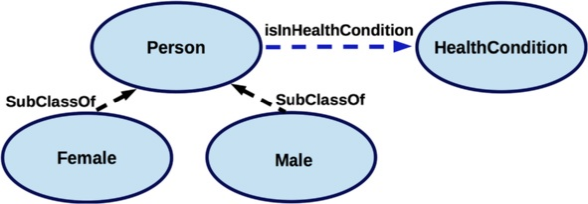
Common module classes. Linking of Person and HealthCondition via the object property isInHealthCondition enables the integration of domain-specific modules used for the gender-specific assessment of health condition

**Fig. 5:**
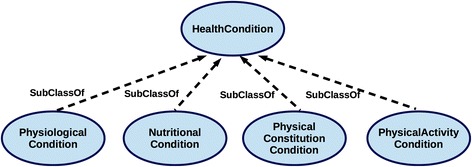
Extending HealthCondition with the key subclasses. The subclasses of HealthCondition are captured in the domain-specific ontology modules (O1-O4); the heterogeneous knowledge-domains relevant for the obesity assessment and prevention are integrated via the subsumption relationship

**Fig. 6:**
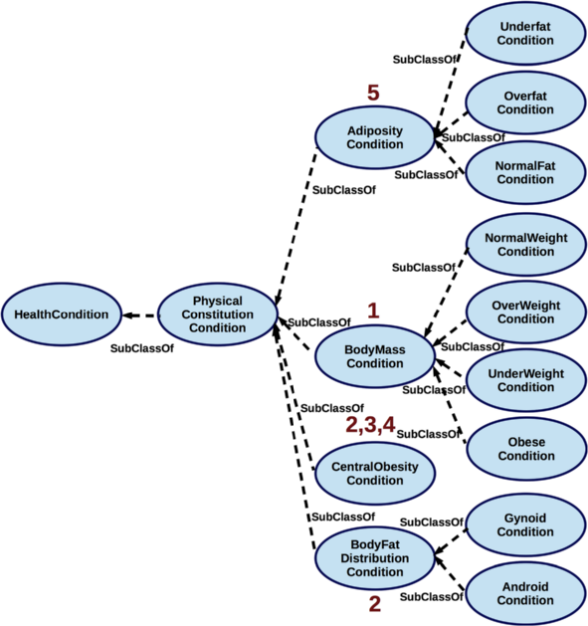
Classifying the obesity-related conditions within the *PhysicalStatus* module. The criteria used to specify the conditions of physical constitution relevant for the obesity assessment distinguish the classes according to 1) Body Mass Index; 2) Waist To Hip Ratio; 3) Waist Circumference; 4) Waist To Height Ratio; and 5) Body Fat Mass

HealthCondition is conceptualised as the most general class capable of capturing diverse physical and behavioural features that describe health-related features of Person. Accordingly, the most general classes of the ontology, i.e. the class HealthCondition and the class Person, are defined together with the most relevant object and data properties in the *Common* module (Fig. 4), i.e. the cross-domain TBox composed of the classes and properties that are common to all the domain-specific ontology modules. While subclasses of Person, i.e. Male and Female, are defined in the *Common* module, the subclasses of HealthCondition are specified in the dedicated domain-specific modules (T1-T5 in Fig. 3). Since the characterisation of a phenotype as *obese* is based on the assessment of one’s physical constitution (weight, height, body mass index, considered in the context of age and gender), the obesity classification is captured within the *PhysicalStatus* module which defines further the subclasses of HealthCondition as the hierarchy of PhysicalConstitutionCondition (see Fig. 6). Likewise, other domain-specific modules define subclasses of HealthCondition (Fig. 5) in an onto-sensitive manner. Thus, PhysicalActivityCondition and its subclasses are defined in *PhysicalActivityBehaviour* module, NutritionalCondition classes in *NutritionalHabits* module, PhysiologicalCondition classes in *PhysiologicalStatus* module. The class HealthCondition of the *Common* module enables the integration of the sub-domain modules via the subsumption relationship (c.f. Figs. 3 and 5). On the other hand, each of the domain-specific modules can be used independently as they consist of domain-specific hierarchies that describe target domains in a comprehensive and exhaustive manner (see e.g. Fig. 6). Besides the hierarchies, each of the modules specifies data properties that define *how* some obesity-related health condition is assessed. In this way, each module explicates formally *evidence* for the assessment of some health condition as based on the current scientific knowledge.

Consider, for instance, the *PhysicalStatus* module as the example on which we illustrate formalisation of the evidence-based assessment of health condition. Figure 6 presents the hierarchy of the relevant health conditions, specifying the physical constitution that considers adiposity, body fat distribution, body mass, and central obesity. Each of the conditions is associated with a specific classification and linked to the reference values that characterise physical features relative to gender and age [40]. These classifications are distinct as they are using diverse criteria to describe a condition of body constitution.

For instance, the criterion of body mass (provided as body mass index [40]) in one of the classifications is used to distinguish people as belonging to one of the following groups: *obese, underweight, overweight* or *normal weight* [25]. According to the classification that considers fat distribution, a person may be classified either as *android* or as *gynoid*. Figure 6 depicts the hierarchy of health condition subclasses based on the description of body constitution via diverse classificatory criteria. Numbers (1-5) in Fig. 6 associated with the classes stand for the classificatory criteria used in *PhysicalStatus* module to characterise the associated condition via the following data properties:

In other words, *central obesity* can be assessed by providing information on either (2) or (3) or (4); *gynoid* and *android* status is assessed based on (2); *obesity* status is assessed as one of *the body constitution classes* as based on (1); *adiposity* status is assessed based on (5).

In addition, the classification is annotated with the reference sources and relevant scientific literature providing evidence for the classificatory choices. Finally, the classification (Fig. 6) captures various types of obesity sub-classifications that are grouped into one (i.e. *PhysicalStatus* module) because all of them satisfy the common criterion of describing phenotype via characterisation of *physical constitution*.

Regarding the above mentioned specialisation of the model to characterise health conditions specifically for the population of *teenagers*, the inference rules, together with the reference values, are defined within the domain-specific sets of SWRL rules. The following subsections present how the ontology is used in practice to personalise, and automatically asses, an obesity-related health condition. The personalised inference is achieved by combining OWL-TBoxes and SWRL-RBoxes that specify inference rules, particularly considering the population of teenagers.

### Combining OWL and SWRL to personalise obesity assessment

SWRL is an expressive DL-based rule language that allows specification of rules expressed in terms of OWL concepts while enhancing the deductive reasoning capabilities [20, 42]. A SWRL rule is structured as a conditional, consisting of an antecedent (i.e. body), and a consequent (i.e. head), as illustrated below with the examples of SWRL rules (see A*x*_1_ - A*x*_10_). SWRL supports only the conjunctive form, and it does not support negated atoms or disjunction [20]. The predicate symbols of a SWRL atom within a rule can include OWL classes, properties or data types. The SWRL arguments can also be OWL individuals, data values or variables. In order to face the undecidability that might accompany the high expressivity of SWRL, we follow the recommendation for the use of DL-safe SWRL rules (see [42], p. 113).

In order to personalise inference about someone’s health condition, the classification of individuals is performed by combining the defined SWRL rules with the OWL declarations that formalise the domain-specific yet generic classifications of health condition and the facts asserted about instances of classes Person and HealthCondition.

**Fig. 7:**
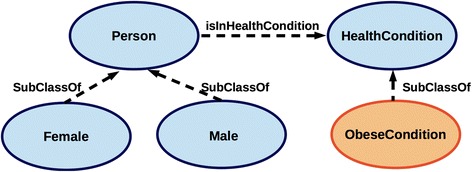
A fragment of the model designed to infer automatically personal health condition

While the domain-specific modules (see T1-T5 in Fig. 3) provide the hierarchies that are utilised in the instantiation of health conditions, the *Common* module (O1) provides the classes used in the instantiation of basic personal information, including the link between the person and their health condition. Precisely, the *Common* module (O1) specifies Person and HealthCondition as two key OWL classes. These two classes are linked by a restriction involving the object property isInHealthCondition (Fig. 7), so that an instance of Person can be associated with one or more health conditions. Specification of physical and behavioural features to characterise *directly health condition* and only *indirectly a person* (via the relationship isInHealthCondition) enables the assignment of diverse health conditions to a person, thus capturing diverse physical and behavioural features of an individual while tracking the evolution of the captured health conditions and associated features over time (the pattern is extrapolated from an analogous approach [43]). In order to assess a specific condition it is necessary to declare the key properties describing a person (see P1) and the time of the condition assessment (H1) that are specified in OWL’s Manchester syntax [44] as the following restrictions:

The inference patterns are modelled further as the rules that classify personal health conditions as belonging to some of the HealthCondition subclasses (see Fig. 6). In other words, the rules identify which class an assessed condition actually belongs to (based on (P1), (H1), and data that characterise the assessed condition, e.g. BMI specified as 1 in (C1)). The reasoning over the classes and inference of a certain condition attributed to a specific person, are performed by means of SWRL rules and Pellet reasoner [45] that makes use of the asserted facts about (1) physical (structural) and functional (metabolic, etc.) features, (2) gender, and (3) the age of a person.

The asserted functional and structural features as well as age are directly associated with a health condition. For example *the body mass index (BMI)* defined via the data property isCharacterizedByBodyMassIndex (see 1 in (C1) and Fig. 6) characterises the health condition of a person that is assessed at specific age (see isAssessedAtAge in (H1)). In particular, BMI is used as the criterion to classify people as being in a health condition that belongs to one of the BodyMassCondition subclasses (Fig. 6). However, BMI is not sufficient to classify a person as being in ObeseCondition or in OverweightCondition. Associating one of the PhysicalConstitutionCondition subclasses with a person requires assertion of the facts that define age and gender of that person [25, 40].

The *gender* is defined by instantiating a Person as belonging to one of its subclasses, i.e. classes Male and Female within the *Common* module (Fig. 7).

The *age* of a person at the time when the health condition is evaluated is crucial information because the reference values for the assessment are particularly variable in adolescence when body grows and changes [40]. In order to capture this variability that can impact on the assessment, the classes Person and HealthCondition are characterised via the object properties and restrictions specified in (P1) and (H1), thus enabling the age-specific assessment of health condition.

Having the data related to the date of birth and time of assessment, we can apply a rule modelled in SWRL [20] in order to get an age-value associated with a personal condition assessment, so that all the needed elaborations can be performed by a reasoning tool without needing to interface with other applications. The rule is specified as

The age calculation rule (A*x*_0_) utilises the SWRL built-ins defined for various numeric types [20]. Such a software-independent age calculation facilitates testing, as shown in the examples that employ rules to infer specific health condition (see Section on the instantiation, Fig. 9).

The following axioms (A*x*_1_ - A*x*_10_) exemplify the sets of SWRL rules that are defined according to the domain-specific criteria used to asses some health condition as based on age and gender. The axioms labelled with the odd subscripts are specifying inference-rules for the male population, while the even-subscript axioms define the rules to classify health conditions associated with female individuals. The examples are just a fragment of the rule sets formalised within the PhysicalStatus RBox (see R1).

Generally speaking, whenever the conditions specified in the antecedent hold, then the conditions specified in the consequent must also hold [20]. The listed rules are structured to specify in antecedent (body) a variable ‘p’ that can have as its extension some of the instances asserted as members of the class Person (specified in OWL) within the *Common* module. The variable ‘h’ should have as its extension members of the class HealthCondition. Age is represented by the variable ‘age’ (calculated in a separate rule, Ax_0_), while gender is specified as a predicate (either Male or Female) associated with ‘p’. For instance, Axiom 10 can be interpreted in natural language as a conditional declaring that for any female person of age between 13 and 17, who is also in health condition that is characterised by waist to hip ratio greater or equal 0.85, we can infer that the asserted health condition of that person is AndroidCondition. In this way the rules lead to new knowledge, thus expanding the Knowledge Base with new information that classifies health conditions associated with persons based on the information describing particular phenotype.

The following subsection presents how the above-introduced classes are instantiated in practice and explains the reasoning steps that exploit specific information at the time of assessment, date of birth, specific characterisation of physical features that are all together used to infer a personal health condition.

### The ontology instantiation and testing

The ontology validation was performed on the test cases designed to capture diverse profiles of teenagers by instantiating classes Person and HealthCondition and assigning data values to the instances in a realistic manner across the domain- specific modules. This section illustrates the employment of the ontology in the reasoning over instances by means of an example of the obesity assessment that provides an explanation of the above-presented design patterns on the application level.

**Fig. 8:**
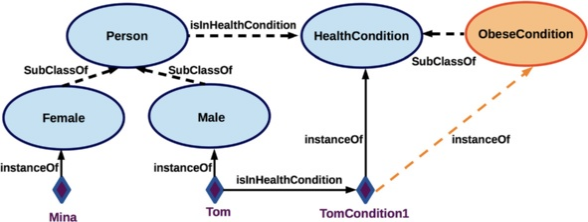
Depicting the reasoning over instances, e.g. the dashed bright arrow (orange) stands for an inferred relationship, automatically classifying TomCondition1 as an ObeseCondition

Figure 8 is an extension of Fig. 7 that exemplifies the instantiation of the classes Person and HealthCondition. In particular, we will focus on the instance representing a boy, named Tom, born in October 2000. By declaring explicitly Tom’s birth data, body mass index (BMI) value and the date of BMI assessment, the ontology enriched with the rules (A*x*_0_ and A*x*_5_) automatically infers that the condition TomCondition1 associated with Tom is an ObeseCondition. This fact presents the new information inferred via ontology which ABox previously contained only the facts about Tom’s birth data, gender, and the data used to characterise his health condition (i.e. BMI).

**Fig. 9:**
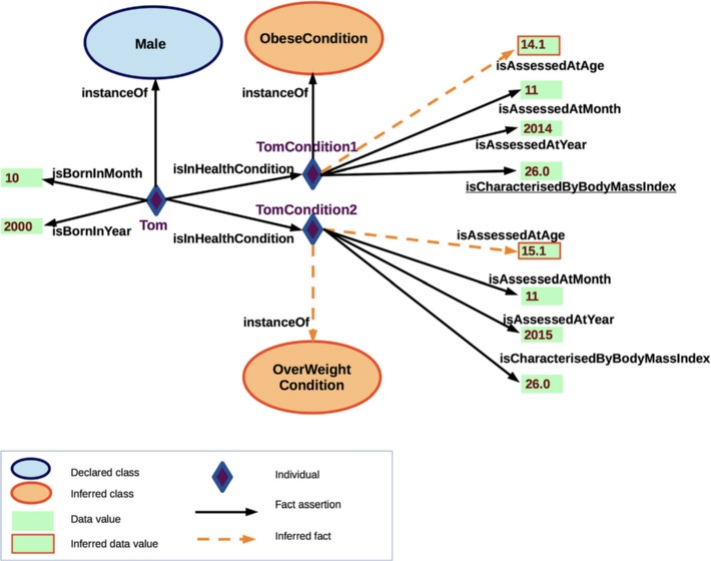
A fragment of the ontology with the example of inference to the personal health conditions at different time points; TomCondition1 is classified as ObeseCondition while TomCondition2 is OverWeightCondition

Since the goal of the ontology is to capture changes of phenotypes associated with a person over time, consider the scenario in which Tom is assigned two assessments of his health condition at two different time points: TomCondition1 (assessed in November 2014), and TomCondition2 (assessed in November 2015) (see Fig. 9). Since the reference value for the assessment of an obese condition changes with age (see the age dependent reference values in A*x*_1_ - A*x*_10_), given that Tom’s age changes while his body mass index stays unchanged, only TomCondition1 is inferred to be *ObeseCondition*, while TomCondition2 is classified as *OverWeightCondition*.

The following specification captures this in OWL’s Manchester syntax [44].

The facts resulting from the reasoning can be saved into the ontology, thus actually enriching the Knowledge Base. The inferred facts can be safely added to the Knowledge Base because the addition of new instances of the assessed health conditions will not invalidate the previous inferences, thanks to the adopted modelling pattern. Moreover, the analysed ontology provides the explanation that Tom’s condition TomCondition2 is assessed as OverWeightCondition in November 2015 based on BMI. On the other hand, the ontology explains that Tom’s ObeseCondition is assessed in November 2014 based on the BMI that characterises TomCondition1.

The same reasoning can be performed with persons of different ages and genders. The domain-specific RBoxes specify the inference rules with the reference values relevant for assessing the obesity-related categories of a person’s health condition (characterised by measure of waist circumference, waist to hip ratio etc. as illustrated e.g. in Fig. 6) by defining a total of 76 SWRL rules specific for the population of teenagers. Inference to health condition based on information about body mass assessment specifies 56 rules; the assessment based on body fat distribu- tion consists of 4 rules; the inference on the presence of central obesity is performed via employment of 8 rules, and adiposity condition assessment also specifies 8 rules. Besides the 76 rules used to classify obesity-related behavioural, physical, and physiological conditions, several rules are used to calculate derived parameters, such as body mass index, age, etc. As a comparison, the work by Scala et al. [18] contains approx. 40 rules, covering only a fraction of the obesity types.

The ontology testing is performed by instantiation of the classes that capture physical, physiological, and behavioural features of individual people (see Figs. 3, 5, and 6) and then running the Pellet reasoner [45] to properly classify the asserted health conditions associated with the instances of persons. Pellet supports inference over the DL-Safe rules and reports on possible errors and misuses of SWRL. The ontology editor Protégé [46] was employed in the creation of TBoxes and RBoxes.

**Fig. 10:**
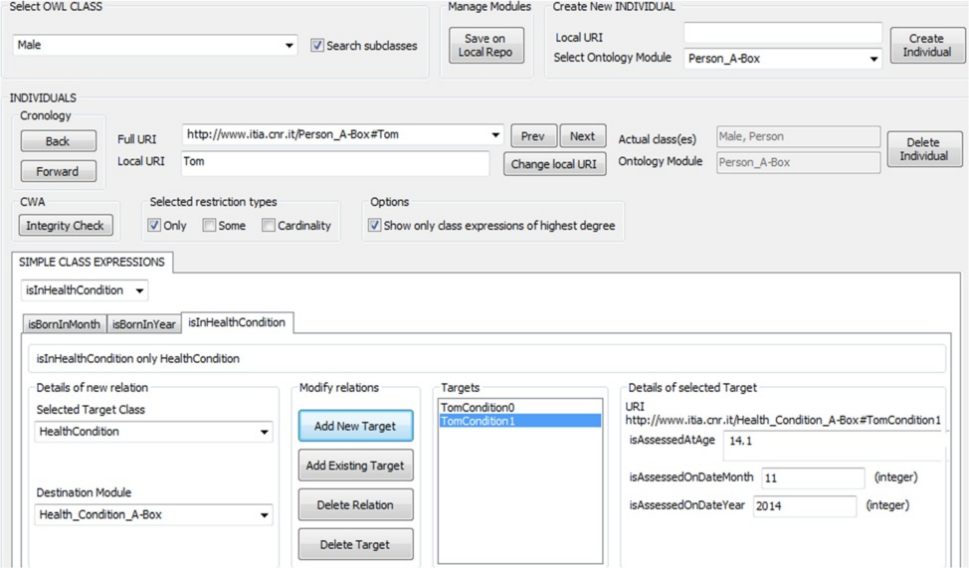
The OntoGui Individual Manager tool instantiates classes Person and HealthCondition, storing the facts about them into dedicated libraries

The ABoxes are managed as separate modules via the OntoGUI tool [47] that, inter alia, supports fast instantiation of ontology Tbox (see Fig. 10). The modules (i.e. libraries generated by OntoGUI) can be used afterwards as inputs for other ontology based applications. The control panel of OntoGUI enables the creation and loading of ontology modules from a repository (either file-based or triple stores) and provides access to several functional modules (see [47]), including *Individual Manager* (Fig. 10) that is a general purpose tool for the exploration, generation and characterisation of OWL individuals. The user interface of the *Individual Manager* is dynamically reconfigured whenever an OWL class is selected. The dynamic reconfiguration is enhanced with the OntoGUI’s capacity to extract information related to the following axioms defined in the Tbox ontology:

- Equivalent classes, both defined as single classes or union of classes;
- Subclasses, both defined as single classes or union of classes;
- Restrictions of any degree if they involve universal quantifier, existential quantifier, or cardinality constraints.

Figure 10 presents the case of instantiation of a subclass of the class Person and assertion of the key facts about the instance via the OntoGUI *Individual Manager*. The facts are automatically stored in the dedicated ABoxes.

### Integrating the privacy concerns and modelling tasks

**Fig. 11:**
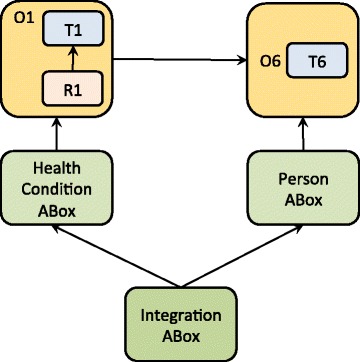
The modularisation pattern that enables (re)use of *a single domain-specific ontology module* (e.g. characterising body constitution); the ABox partitioning supports protection of personal data

**Fig. 12:**
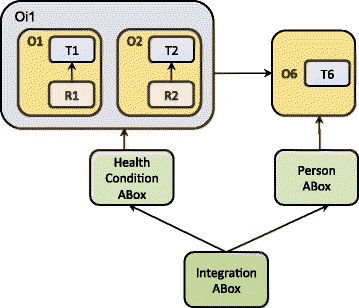
The modularisation pattern that enables (re)use of *two domain-specific ontology modules*, O1 and O2, integrating them with the partitioned ABox that supports protection of personal data

**Fig. 13:**
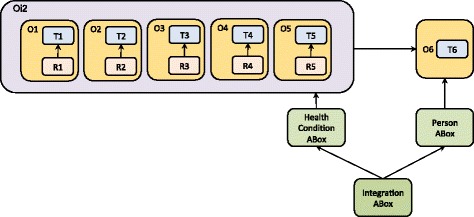
The modularisation architecture that *integrates multiple domain-ontologies* and supports protection of personal information via the ABox partitioning

The above-presented ontology can capture physical and behavioural features of individuals related to their health condition that include sensitive information. Accordingly, the Knowledge Base (KB) [48, 49] architecture was designed specifically to support management of personal information by means of the ABox modularisation (Figs. 11, 12 and 13). Besides the TBox and RBox modules (Fig. 3), the KB consists of the ABox modules dedicated to the assertion of facts about instances that represent individual people and their health status. More specifically, the ABox is partitioned into:

- *HealthCondition ABox* containing instances that represent the target health conditions and facts about them, i.e. criteria used to characterise the assessed condition;
- *Person ABox* containing instances that represent persons and basic facts about them, i.e. gender and date of birth;
- *The Integration ABox* that imports *Person ABox* and *HealthCondition ABox* and contains assertions of the links between the facts stored within the separate ABoxes.

Accordingly, access to the stored data can be managed separately on each of the three levels. Any assertion of the facts about instances requires a concurrent access to both ABoxes that will only jointly associate some health status with appropriate instances representing persons. The partition of ABoxes is motivated by ethical concerns and it aims to support privacy of the health-related data also on the modelling level.

Figure 13 depicts the complete KB architecture, including the relationships between the ontology modules: RBoxes import the dedicated TBoxes that in turn provide terminology for the reasoning rules formalised in RBoxes. ABoxes on the other hand import directly RBoxes and indirectly domain-specific TBoxes and *Common* TBox, thus enabling the merging of sub-domain ontologies into the integrated and populated ontology. Alternatively, the modular structure enables independent development, employment, re-use, and evolution of the domain-specific segments. For example, Fig. 11 presents only one fragment of the ontology presented in this paper (O1), i.e. the ontology that captures obesity-related knowledge focused on *physical constitution* (see Fig. 6). If the modelling task changes to include the information related to *physical activity* and integrate it with the model that represents physical constitution, the relevant modules will be imported and integrated accordingly (see Fig. 12).

Figures 11, 12 and 13 together exemplify a variety of the modes to (re)use the ontology-modules, where particular modules are employed according to the demands of the modelling task that might be focused on data collection, data retrieval, and/or reasoning over some of the obesity-related domains such as personal body constitution, physical and nutritional behaviour, etc.

Nonetheless, independently of the modelling task, the module-integration patterns *conserve the links*

- between the *Common* module (O6) and *Person ABox*;
- between the domain-specific ontology modules (O1-O5) and the *HealthCondition ABox*.

In other words, the domain-specific ontology modules (O1-O5) are used to classify individual health conditions, while the assertions of the facts about the conditions are stored in the corresponding *HealthCondition ABox*. The integrated ABox brings together personal information and generic obesity-related knowledge (captured as the OWL classes (T1-T5) and SWRL reasoning rues (R1-R5)) as it stores both asserted and inferred facts, including the links that hold between instances of the classes Person and HealthCondition.

## Conclusions

This paper described the ontology that captures several obesity-related knowledge-domains: Physical Status Domain, Physical Activity Behaviour-Domain, Physiological Status Domain, and Nutritional Habits Domain. The ontology is designed to support flexible use and reuse of captured information, the interoperability between technological devices, the integration of collected information, and automated inference about personal status over time. The modular structure is adopted in order to enable independent development, maintenance, evolution, and validation, as well as the integration of diverse domain-specific modules. The modular design enables the use and combination of the modules according to the needs of a particular task, while each of the modules can be used separately from others and the ontology can be extended with new modules that can be added at a later stage.

Besides the validation and domain-coverage criteria, the modularisation methodology included the criterion that distinguishes disciplinary perspectives, the meta-topic criterion, and the integrative-view criterion. In particular, the *Common* ontology module was developed to support the integration and interoperability between the domain-specific modules as well as tracking the evolution of personal health condition over time. The combination of two formal languages motivated partition of the modules into TBoxes specified in OWL and RBoxes specified in SWRL, while the ethical concerns motivated partition of the ABox into the segments that store separately facts about persons and those about health conditions.

In particular, the paper illustrated how health conditions are associated with the physical constitution (i.e. obesity-related) classes, and are then employed to infer automatically personal health status as age- and gender-dependent.

The forthcoming task is to perform the ontology testing within a Semantic repository that will be developed and populated with real data (i.e. instances representing adolescents and their phenotypic features) acquired through the pilot studies of the PEGASO project ([23] p. 19). In terms of databases integration and interoperability, the activities will include mappings between the ontology and several task-oriented databases developed to store the data acquired from wearable devices, nutrition-related questionnaires etc. The research related to the exploitation of a Semantic repository (see e.g. [50]) will have to deal further with the compatibility between the ontology and available technological solutions (e.g. Stardog [51]) that add certain modelling constraints in terms of supported OWL2-profiles [52].

Regarding the interoperability and integration with other ontologies, future work will examine possible links and alignments [32, 53] with the relevant phenotype ontologies [54, 55], the reference terminologies and ontologies [56–62], as well as the foundational ontologies [63–66]. The references to certain standards, e.g. LOINC’s definitions and codes [67] are currently present only in the annotation of some of the represented concepts and additional work is required concerning the ontology annotation.

The further ontology development will include additional specification of the relevant information, e.g. the reasoning about the behavioural patterns and interventional strategies, the formal specification of other relevant phenotypic features and target behaviours relevant for the motivation of healthy lifestyle.

The presented ontology primarily considers obesity prevention and not obesity-related diseases. However, some health conditions (e.g. diabetes, some food type intolerances, etc.) require abstention from certain types of food that would otherwise be recommended as beneficial to any other individual who is not diagnosed with the condition. Thus, the model of an interventional strategy regarding nutrition should explicate the information about particular disease-related health conditions that might impact the interpretation of the concept *Healthy food*. Extending the ontology with the module that makes explicit links with diseases is of a high relevance and the existing ontologies (e.g. [18]) will be examined in this respect as the candidates for the ontology integration and re-use. In addition, making an extrapolation from the local community models [2, 17] is the initial ground for the development of a new module that would capture the healthcare policy domain on a generic level. The module that represents various stakeholders and decision makers involved in the prevention and management of obesity could help to explicate normative aspects of interventional strategies that often stay implicit in the models that target only limited fractions of scientific knowledge.

## Abbreviations

BMI: body mass index
KB: knowledge base
OWL: web ontology language
SWRL: semantic web rule language
